# Comprehensive framework for visualizing and analyzing spatio-temporal dynamics of racial diversity in the entire United States

**DOI:** 10.1371/journal.pone.0174993

**Published:** 2017-03-30

**Authors:** Anna Dmowska, Tomasz F. Stepinski, Pawel Netzel

**Affiliations:** Space Informatics Lab, Department of Geography, University of Cincinnati, Cincinnati, OH, United States of America; Northwestern University, UNITED STATES

## Abstract

The United States is increasingly becoming a multi-racial society. To understand multiple consequences of this overall trend to our neighborhoods we need a methodology capable of spatio-temporal analysis of racial diversity at the local level but also across the entire U.S. Furthermore, such methodology should be accessible to stakeholders ranging from analysts to decision makers. In this paper we present a comprehensive framework for visualizing and analyzing diversity data that fulfills such requirements. The first component of our framework is a U.S.-wide, multi-year database of race sub-population grids which is freely available for download. These 30 m resolution grids have being developed using dasymetric modeling and are available for 1990-2000-2010. We summarize numerous advantages of gridded population data over commonly used Census tract-aggregated data. Using these grids frees analysts from constructing their own and allows them to focus on diversity analysis. The second component of our framework is a set of U.S.-wide, multi-year diversity maps at 30 m resolution. A diversity map is our product that classifies the gridded population into 39 communities based on their degrees of diversity, dominant race, and population density. It provides spatial information on diversity in a single, easy-to-understand map that can be utilized by analysts and end users alike. Maps based on subsequent Censuses provide information about spatio-temporal dynamics of diversity. Diversity maps are accessible through the GeoWeb application SocScape (http://sil.uc.edu/webapps/socscape_usa/) for an immediate online exploration. The third component of our framework is a proposal to quantitatively analyze diversity maps using a set of landscape metrics. Because of its form, a grid-based diversity map could be thought of as a diversity “landscape” and analyzed quantitatively using landscape metrics. We give a brief summary of most pertinent metrics and demonstrate how they can be applied to diversity maps.

## Introduction

Census Bureau population projections [1] indicate that the racial dynamic in the U.S. will steer the country toward a society with no absolute racial majority by 2044. How this overall prediction translates to a change in the racial makeup of local neighborhoods is of great interest to academics, as well as to policy makers and to the general public. Racial makeup is quantitatively analyzed in terms of *segregation* or *diversity*. A classic definition of racial segregation is the physical separation of two or more groups into different neighborhoods [2]. From a spatial point of view, segregation is the spatial pattern reflecting varying contributions of two races to local sub-populations. When the population is multi-racial rather than bi-racial, its makeup is studied in terms of diversity rather then segregation; diversity is a spatial pattern reflecting varying contributions of all races to local sub-populations. Analysis of segregation and/or diversity capable of yielding results which are lucid and useful to all stakeholders is important for a better understanding of the processes responsible for the observed segregation/diversity and to better inform the decision makers.

There is a large body of scientific literature on how to analyze racial segregation in the U.S. starting from the 1960s [3] when the focus was on Black/White segregation. A lot of studies were undertaken after the release of U.S. Census data in the year 2000 [4–13]. More recent studies include [14–19] and [20]. Interestingly, a significant portion of this literature is devoted to addressing shortcomings of existing methods of analysis (for example, [7, 21, 22]) and to proposing new methods of analysis (for example, [17, 23, 24]). The fact that this trend continues for four decades indicates an enduring weakness of the methodology. A common thread to all proposed methods of analysis is their reliance on data format (areal unit-based aggregation of population counts) provided by the U.S. Census Bureau data. In other countries segregation studies [21, 25–27] are also based on the same data format. As methods of analysis are constrained by the format of the data it is reasonable to hypothesize that the unsatisfactory state of the methodology is, at least in part, due to limitations of data format [22].

The Census releases demographic data aggregated to areal units—also referred to as small areas or statistical areas; hereafter we will refer to them as Census units. Aggregations to units of different sizes are available starting from blocks (the smallest) to states (the largest). Most segregation studies utilize tracts which are the third level of aggregation. There is a growing awareness about the limitations of aggregated data (for an overview see [28]). Several attempts to mitigate these limitations have been proposed [27, 29–31], but only few [17, 24] actually involved changing the format of the input data to grid. We review the shortcomings of aggregated data and argue that a high resolution population grid provides a data format which is more suitable for diversity studies, especially studies aiming at spatio-temporal dynamics of diversity over the entire U.S. We then proceed to describe the process of obtaining a database consisting of high resolution population grids, and introduce SocScape—a freely available online resource for the distribution and visualization of gridded 1990–2000–2010 population data over the entire conterminous United States (CONUS) at the nominal resolution of 30 m. SocScape enables grid-based studies of diversity (including temporal changes) not only for metropolitan areas but also for any region within CONUS.

An overwhelming majority of studies use various indices to describe the state of segregation/diversity. However, as we mentioned above, racial diversity should be analyzed as a spatial pattern and spatial patterns are notoriously difficult to quantify. The most widespread method of quantifying spatial patterns of categorical variable is to use a (large) set of landscape metrics [32, 33]—algorithms that quantify various aspects of the pattern. Although metrics are useful for quantitative comparison of two patterns (landscapes), they are not very effective in conveying an overall character of a single pattern or, in our case, a character of racial condition within an area of interest. For this, a visualization, in the form of a single map, is the most effective way of converting diversity data into actionable knowledge [34] that can be utilized not only be researchers but also by end users.

With this in mind our database also includes a set of diversity maps. A diversity map is a product that we derived by classifying population into diversity/dominant race communities. We demonstrate how diversity maps, which are immediately viewable and/or downloadable from SocScape, provide insight on racial conditions in an area of interest and on their spatio-temporal evolution over the decades of 1990-2010. Through these maps all aspects of racial diversity are observable and, unlike segregation/diversity information in the form of tables of indices, informational content of diversity maps can be appreciated by end users even if they are not specialists in the field.

Finally, we noticed that a diversity map has a format (although not a content) which is analogous to the format of a land cover map. By taking advantage of this analogy the value of diversity maps may be extended beyond visualization as they can be also quantitatively analyzed using well-known and well-developed concept of landscape metrics (see above). We provide a brief description of landscape metrics and demonstrate how to use them to quantitatively analyze racial conditions using a diversity map as an input.

## Shortcomings of aggregated population data

The Census collects race data at the ultimate resolution of an individual household (individual level). In Geographic Information System (GIS) terminology such data is referred to as point data. However, this data is not released to the public in its original format due to privacy concerns. In some other countries, most notably in Sweden, individual level data is made available to scientific organizations under strict supervision [27], but this is not the case in the U.S. There are two GIS formats in which the Census could release the data, vector (shapefile) which aggregates point data to population counts within Census units, or raster (grid) which models the point data to obtain cell-based population density. For predominantly historical reasons [28] the Census releases data in aggregated (vector) format. As this has always been the case, this format may appear as the natural choice for providing information about population characteristics, but, in reality, it has multiple shortcomings in comparison to the grid format. Table 1 summarizes the major shortcomings of the aggregated format of population data and contrasts them with the advantages of using gridded data.

**Table 1:** Population data: Census units versus grid.

	ELEMENT	DATA AGGREGATED TO UNITS	GRIDDED DATA
1	variable	population counts within macro-defined Census units	population density at micro-defined cells organized into a regular grid
2	GIS formats	vector (shapefile) + attribute table; difficult to work with large shapefile files	raster; easy to work with large raster files
3	spatial resolution	dependent on the choice of Census units and spatially varying; low in rural areas	high and spatially constant; defined by the size of the cell
4	uniformity	mapped population appears (incorrectly) to be distributed uniformly within each Census unit	mapped population density changes continuously from cell to cell
5	boundaries	mapped population appears to be discontinuous at the boundaries between Census units	mapped population density changes smoothly between grid’s cells
6	modifiable areal unit problem	statistics depend on the size (blocks, tracts etc.) of Census units	not applicable
7	temporal change	when assessing population change between different years interpolation is needed because the extents of Census units change with time	constant grid enables direct cell-to-cell temporal comparison
8	user-defined areas	to work with user-defined areas (for example, with ZIP Code areas or school districts) interpolation from Census units is needed	populations of user-defined areas can be aggregated directly from grid’s cells
9	neighborhood	neighborhoods are often assumed (incorrectly) to coincide with census tracts	neighborhoods emerge from spatial distribution of population density

The spatial resolution of Census units is variable and low, except in the most densely populated urban areas (the average area of a 2010 census tract is 108 km^2^, but the average area of a rural tract is 209 km^2^, and the average area of an urban tract is 2.7 km^2^). As a result the spatial precision of statistical analysis changes from location to location making comparison between different locations questionable. In particular, any comparison in patterns of diversity between urban and rural areas may be grossly inaccurate. Analysis also suffers from the Modifiable Areal Unit Problem (MAUP) [35, 36] which arises from the imposition of artificial Census units on the continuous distribution of population. Mapping segregation or diversity using Census units results in maps (for example, see [14] or [37]) that suggest spatial uniformity across each unit and discontinuity at the boundaries between the units. This is an artifact of aggregated data and the visual manifestation of the MAUP. Additionally, there is a spatial mismatch [38] between census areal units (blocks, tracts etc.) and user-desired units (for example, neighborhoods, tax zones, postal delivery zones, vegetation zones, watersheds, etc.). Finally, the boundaries of census aggregation units change from one census to another, making the analysis of population change difficult [39–41].

**Fig 1:**
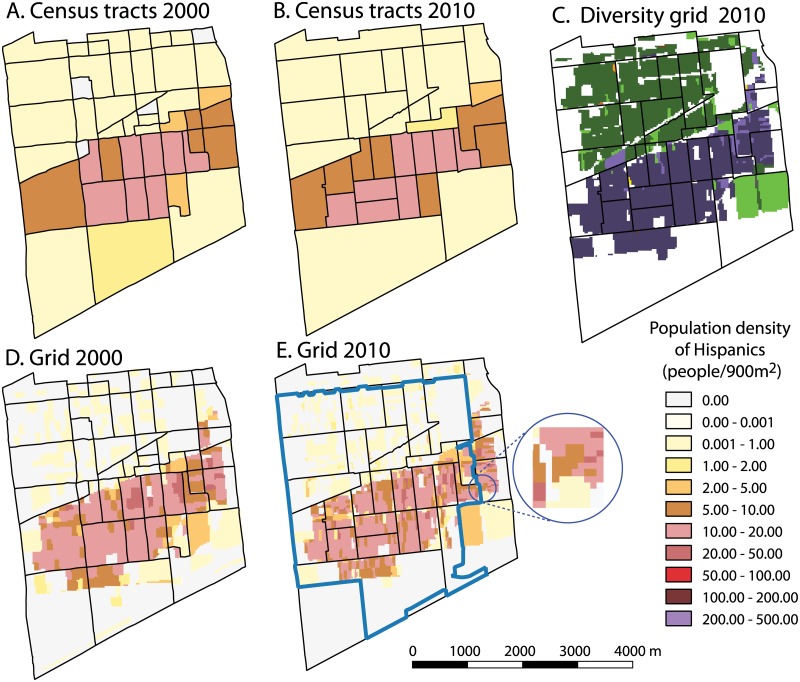
Maps showing distribution of the Hispanic population in central Chicago according to Census tracts (panel A for 2000 and panel B for 2010) and according to high resolution grids (panel D for 2000 and panel E for 2010). Census tracts boundaries are overlaid on grid-based maps for reference. Extent of the ZIP Code is shown in blue. Inset in panel E is to show the high (30m) resolution of the grid. Racial diversity grid-based map (D) shows spatial extents of two communities, Blacks-dominated (green color) and Hispanic-dominated (violet color).

Fig 1 provides visual illustrations for the shortcomings of aggregated data as summarized in Table 1 and discussed in the previous paragraph. It shows a series of maps of an area located in central Chicago roughly restricted by the following streets: North—W. Roosevelt Rd, South—Stevenson Expy S, East—S. Western Ave, West—S. Cicero Ave. Panels A and B show maps of the density of the Hispanic population based on data aggregated to 2000 and 2010 Census tracts, respectively. Panels D and E show maps of the density of the Hispanic population based on 2000 and 2010 high resolution population grid, respectively. Panel C shows a map of racial diversity based on the 2010 grid.

First, notice that the boundaries of the tracts have changed (panels A and B); the area consisted of 47 tracts in 2000 but only 33 tracts in 2010. Thus tract-by-tract comparison is not possible unless data from 2000 is interpolated to 2010 boundaries [39–41], or a commercial product (with interpolation already performed), such as normalized data from Geolytics (http://www.geolytics.com) is used. There is no such problem for grids where direct cell-to-cell comparison can be made (panels D and E). Second, notice that tract-based maps incorrectly show the density of the Hispanic population to be uniform within each tract (panels A and B) but, in reality, density changes within tracts as correctly depicted by the grid (panels D and E). Also, tract-based maps suggest abrupt changes in density at the boundaries between some tracts where there is actually a smooth transition. Third, notice that ZIP Code indicated by the blue polygon shown in panel E cuts trough several census tracts, making aggregation of the Hispanic population within this ZIP Code from tract data impossible without interpolation. On the other hand, Hispanic population of this ZIP Code can be calculated directly from the grid. Finally, notice from panel C that the area is divided into three zones, where the north is dominated by the Black population (green colors), the middle is dominated by the Hispanic population (violet colors), and the south is mostly uninhabited. Although most tracts in this area consist only of one type of community (either Black dominated or Hispanic dominated), some tracts have segments which are inhabited by different communities. Thus, tracts cannot be assumed to have homogeneous communities. The grid-based diversity map (panel C) clearly shows division of the area into the two different communities but this division does not coincide perfectly with tract boundaries.

## High resolution grids

The advantages of gridded population data over aggregated data are purely academic in the absence of complete and accurate gridded data. Until now no such data has been available. The Socioeconomic Data and Application Center (SEDAC) (http://sedac.ciesin.columbia.edu/) provides 1 km resolution (250 m for selected metropolitan areas) demographic grids. However, in addition to having a resolution which is too coarse for segregation studies and being the result of an oversimplified gridding model (areal weighting), these grids are only available for the years 1990 and 2000. A higher resolution (90 m) US–wide demographic grid, presumably based on the most recent Census data, is under development by the Oak Ridge National Laboratory [42]. This project, called LandScan–USA, aims at providing both nighttime (residential) as well as daytime population densities, but it is not currently available, nor is it expected to be in the public domain once it becomes available.

Since 2014 we have been developing [43–45] precise high resolution demographic grids for the entire CONUS. For the purpose of racial segregation/diversity analysis we have developed three types of grids: total population grids, racial sub-population grids and racial diversity classification grids. All grids have 30 m resolution and are the result of dasymetric modeling. Dasymetric modeling (for a review see [46]) refers to the process of disaggregating population data from Census units to a finer grid using ancillary data that correlates with population density but which has a higher resolution [47]. Dasymetric modeling is the most advanced method of population distribution modeling because it utilizes additional information contained in the ancillary data. The original, and still the most widely used, ancillary data are land cover/land use data [47–50].

The ancillary data available for the entire CONUS are 1992, 2001, and 2011 editions of the National Land Cover Database (NLCD). NLCD (http://www.mrlc.gov/), which has a 30 m resolution, maps land cover into 16 categories. It can be utilized as ancillary data for dasymetric modeling because there is a statistical relation between these categories and population density [45]. Additional ancillary data that can be utilized for CONUS-wide dasymetric modeling is the newly available National Land Use Dataset (NLUD2010) [51] which also has 30 m resolution. The challenge in developing multi-year demographic grids via dasymetric modeling is to use compatible multi-year ancillary data. Therefore, we have developed two families of grids.

The first family of grids pertain only to 2010. The source of the demographic data is the 2010 decenial Census block-level data (summary file 1 data, P5 table). To calculate these grids we use a dasymetric model [45] based on the combination of NLCD2011 and NLUD2010. These are the most accurate grids and they should be used for all analysis that do not require assessment of temporal change.

The second family of grids (referred to as multi-year compatible or MYC grids) pertain to 1990, 2000, and 2010. The source of the demographic data is the 1990, 2000, and 2010 decenial Census block-level data (SF1 P5 table for 2010, SF1 P8 table for 2000, and STF1 P10 table for 1990). These grids have been developed to be year-to-year compatible and thus should be used for analyzes that require assessment of temporal change. For this reason NLUD (available only for 2010) is not utilized in calculating these grids. Furthermore, because of the incompatibility between the legend for NLCD1992 and the legend for later editions of NLCD, we don’t use the complete set of NLCD categories as ancillary data, instead we use NLCD2011 and 2001 reclassified to three categories (urban, vegetation, uninhabited) and NLCD 1992/2001 Retrofit Land Cover Change Product [52] also reclassified into the same three categories. Thus, the 2010 MYC grid is a little less accurate than the 2010 grid calculated using the combination of NLCD and NLUD, but is backward compatible.

### Race/Ethnicity grids

Because land cover/land use categories correlate only with the total population count and not with its race-based sub-populations, we use dasymetric modeling to disaggregate the total population count. Disaggregation weights—numerical coefficients calculated to enable disaggregation—are stored and used to disaggregate race-based sub-populations. Thus, the high resolution grid of race-based sub-populations is not accounting for possible sub-block inhomogeneities in the proportion of each population segment to the total population, but it gives a more accurate spatial location of each sub-population by keeping it away from uninhabited areas and making an adjustment in line with the sub-block overall population density.

We calculated high resolution grids for seven race/ethnicity sub-populations: non-Hispanic whites (NHW), non-Hispanic blacks (NHB), non-Hispanic Asians (NHAS), non-Hispanic American Indians (NHAM), non-Hispanic Native Hawaiians and Other Pacific Islanders (NHPI), non-Hispanic other races (NHO), and Hispanics (H). For each grid cell, the summation over values of densities for all sub-populations yields the density of the total population assigned to this cell. The summation of densities (of either the total population or any sub-population) over all grid cells in a census block recovers the corresponding Census count for this block.

### Diversity map

In addition to race/diversity grids we also make available a 1990-2000-2010 set of diversity grids. A diversity grid is a product derived from race/ethnicity grids that maps the patchwork of areas inhabited by different diversity/race-defined communities. It is important to stress that we use the word “community” to indicate a class or category of population (as well as a spatial domain where they live) defined exclusively by their racial mix. This may or may not coincide with the notion of community as a spatial unit grouping people with similar social identity and practice. Diversity map is particularly useful for visualizing (in high spatial resolution) spatial configuration of overall racial conditions anywhere in the CONUS. As such, it may prove to be the only diversity information most users need, especially because it could be easily explored and acquired by using the online application SocScape. This application also allows for visual assessment of segregation/diversity change between 1990, 2000, and 2010 by switching between layers of information for different years.

The idea of classifying population into groups based on diversity and dominant race has been proposed before but was applied mostly to Census tracts. In [25] a scheme to classify tracts into six categories depending on the relative abundance of their dominant group and two other groups was presented. In [9] the authors classified tracts in 10 largest U.S. metropolitan statistical areas (MSAs) during the 1990s into seven types based on diversity and dominant race. In a similar study, but extended to 100 largest US MSAs, [13] classified racial structure within tracts using a scheme based on diversity and dominant race. A very similar classification scheme was used by [14] to assess and visualize the 1990-2000 change in diversity in sixteen large US MSAs. Most recently [20] categorized and mapped neighborhood transitions between 1970 and 2010, and [17] clustered neighborhoods (tracts) in Southern California into 20 categories based on diversity, dominant race, and scale. Subsequently, they visualized the spatial extents of these clusters on the map. These works demonstrated that classification of communities (neighborhoods, tracts, etc.) is the most effective way to map racial conditions in a given area and to visualize how they change over time. We extend this approach to the classification of cells in a high resolution grid over the entire CONUS.

To produce a racial diversity grid we assign each cell in a grid one of 40 possible labels; 39 of these labels correspond to different communities and the 40th label corresponds to uninhabited areas. Our classification follows the method introduced by [14] but adds an additional discriminant—population density, because even with the same level of diversity and dominant race communities having vastly different population densities have different characteristics. This distinction is particularly important when comparing urban and rural areas. Thus, our classification is based on three discriminants: dominant race, racial diversity, and population density.

Density values for NHW, NHB, NHAS+NHPI, NHAM, NHO, and H at each cell are collected. If the total density at the cell is equal to zero, the cell is assigned an *uninhabited* label. Otherwise, the cell’s dominant race is the one with the largest value of density. Notice, that we have combined NHAS and NHPI because in Census 1990 these two groups were combined. The degree of racial diversity is measured by a combination of standardized informational entropy *E* [53] and the percentage of the dominant race calculated for each cell from race/ethnicity grids. The value of *E* is calculated from the values of the six race-specific densities normalized to add up to 1. Diversity is classified into three categories: low, medium and high. Diversity is *low* if *E* < 0.37 and the percentage of the dominant race is > 80%, and it is *high* if *E* > 0.73 and the percentage of the dominant race is < 50%. All remaining inhabited cells are classified as having *medium* diversity. Population density is classified into three categories; *low*—having values of < 3 people/km^2^, *medium*—having values of 3–30 people/km^2^, and *high*—having values of > 30 people/km^2^.

**Fig 2:**
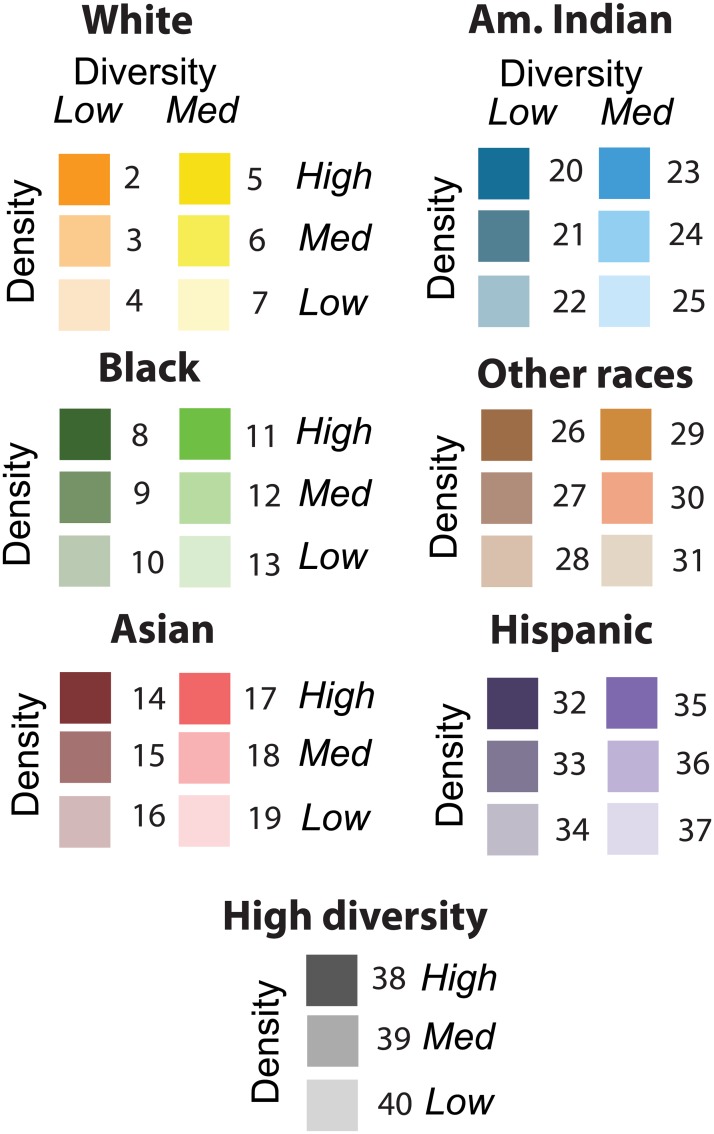
Classification of population into 39 different communities based on degree of diversity, dominant race, and population density. Colors serve as a legend to the diversity maps (see Fig 3), numbers are numerical labels as encoded in diversity data downloadable from SocScape.)

Combining dominant race with diversity categories and population density categories we obtain 39 different categories of communities. This is because the high diversity category does not need to be sub-labeled by a dominant race. Fig 2 illustrates how the three different factors (dominant race, diversity, and population density) combine to form 39 categories of communities. We assign a color (to be used in mapping) and a number (for quantitative analysis) to each community. Note that if distinction between different population densities is not needed or desired, the classification can be collapsed (by means of reclassification) to just 13 diversity/dominant race categories plus an uninhabited category.

## Data accessibility and availability

All our grids (total population, race sub-populations, and racial diversity) are freely available for download. Data can be accessed by two different methods: (1) using our GeoWeb mapping application SocScape, and (2) using county-by-county or MSA-by-MSA download. Table 2 summarizes what gridded data is available for download using the two methods. It also provides detailed information on original Census data used in our models.

**Table 2:** Availability of grid population data.

	NHW	NHB	NHAS	NHAM	NHPI	NHO	H	Total	RD
**1990**	P010_003	P010_004	P010_006	P010_005		P010_007	P010_008	P000_001	
SocScape								✓	✓
Counties	✓	✓	✓	✓		✓	✓	✓	✓
MSA	✓	✓	✓	✓		✓	✓	✓	✓
**2000**	P008003	P008004	P008006	P008005	P008007	P008008 + P008009	P008010	P001001	
SocScape								✓	✓
Counties	✓	✓	✓	✓		✓	✓	✓	✓
MSA	✓	✓	✓	✓		✓	✓	✓	✓
**2010**	P0050003	P0050004	P0050006	P0050005	P0050007	P0050008 + P0050009	P0050010	P0010001	
SocScape								✓	✓
Counties	✓	✓	✓	✓		✓	✓	✓	✓
MSA	✓	✓	✓	✓		✓	✓	✓	✓

### SocScape

We submit that the most effective medium to convey information about racial conditions is a map showing the spatial extents of different communities. We have constructed such a grid-based map for the entire CONUS at 30 m resolution. The only practical means of viewing this map is through a GeoWeb application. SocScape (Social Landscape) (http://sil.uc.edu/webapps/socscape_usa/) is our internet-based application for exploration of diversity and total population distributions. Maps of individual race-based sub-populations are not available through SocScape. SocScape is an ideal tool to visualize racial conditions anywhere in the CONUS and to observe how these conditions change with time. Because SocScape uses high resolution gridded data, diversity and its temporal change can be observed down to the street level. A street map is also included in SocScape, thus, by using an opacity tool, geographical information can be overlaid on diversity information.

Using the download tool a user can select an area of interest (having a rectangular shape) and download data for this area. Data is downloaded in zipped geoTIFF format. It needs to be unzipped before it can be opened in GIS software such as ESRI ArcGIS (commercial software) or QGIS (free and open software) (http://www.qgis.org/en/site/). The diversity grid, being a categorical raster, opens in ArcGIS and QGIS with the same color assignment as is seen in SocScape. The total population grid, being a numerical raster, opens as a grayscale. Note that population density maps in SocScape are classified into 11 density categories for visualization purposes and given in units of people/km^2^, but downloaded data is unclassified and in units of people/cell.

### County-by-county and MSA data

The complete set of all gridded data (total population, race-based sub-populations, and racial diversity) for all available years (1990, 2000, and 2010) for all counties in the CONUS, as well as for 363 MSAs is available to download from our web page at http://sil.uc.edu/cms/index.php?id=socscape-data. The downloaded file is a zip archive that contains three directories: (1) total population—contains population grids for each year (4 grids) in units of people/cell, (2) diversity—contains racial diversity classification grids for each year (4 grids), (3) race-based sub-populations—contains separate grids for 7 race/ethnicity groups for each year (27 grids) in units of people/cell. All grids are in geoTIFF format. Note that the NHPI grid is not available for the year 1990 because it was included as an Asian sub-population in the 1990 Census.

## An example of using the diversity grid

**Fig 3:**
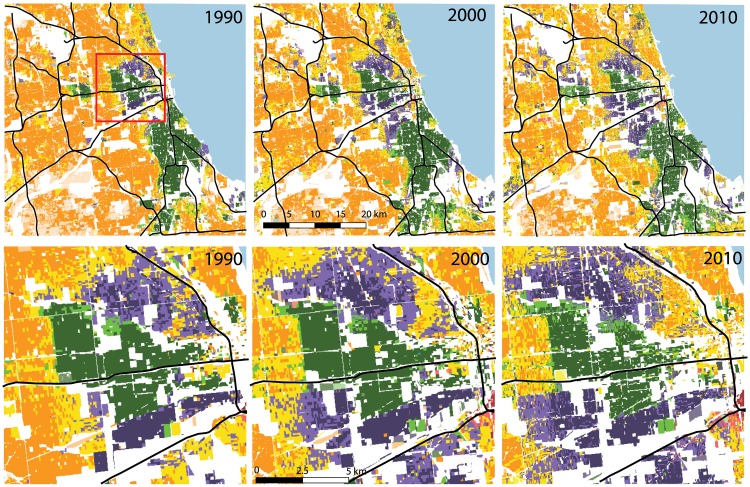
Racial diversity maps of the Chicago area for 1990, 2000, and 2010. The upper row shows maps for the greater Chicago area and the lower row shows maps for the central Chicago area indicated by a red rectangle on the broader extent map for 1990. Major roads are overlaid for geographical reference. For the legend of community categories see Fig 2, the white color indicates uninhabited areas.

To demonstrate the process of analyzing racial diversity using a diversity grid, we use the greater Chicago area as an example. Using the download tool in SocScape we selected an area as shown in the upper row of Fig 3 and downloaded MYC diversity maps for this area for 1990, 2000, and 2010. These maps open in GIS software without any additional processing; they are grids containing 1788 × 1749 cells; each cell’s color corresponds to a specific community (see Fig 2 for legend). To better show details we also enlarged the central area of Chicago and displayed the enlarged maps in the lower row of Fig 3.

We submit that these maps provide lucid and useful information about racial conditions in Chicago and about their spatio-temporal evolution. In particular, notice that they provide visual insight on all five aspects of segregation suggested by [2], and they do this for all racial groups simultaneously. Evenness—the degree to which groups are distributed proportionately across a city—is addressed by a number of different communities and their spatial distributions; we can see that in the Chicago area the degree of evenness is small. Exposure—the extent to which members of different groups share common residential areas—is addressed by the definitions of communities; only high diversity, and, to a smaller degree, medium diversity communities, have a significant degree of exposure. Concentration is the degree of a groups’ agglomeration in urban space; large concentration is seen on the map as large areas of the same color; a high degree of concentration is observed in the Chicago area. Centralization, the extent to which group members reside toward the center of an urban area, and clustering, the degree to which minority areas are located adjacent to one another, are also directly observable on the map.

To better appreciate the usefulness of diversity maps, a reader should momentarily forget the content of Fig 3 and try to arrive to an opinion about diversity conditions in Chicago based on a series of traditional indices. We have calculated these indices from about 1300 (depending on the year) Census tracts coinciding with the area shown in Fig 3. Dissimilarity indices are as follows, ${\text{D}}_{1990}^{B}\;=\;0.86$, ${\text{D}}_{1990}^{H}\;=\;0.63$, ${\text{D}}_{2000}^{B}\;=\;0.85$, ${\text{D}}_{2000}^{H}\;=\;0.62$, ${\text{D}}_{2010}^{B}\;=\;0.80$, ${\text{D}}_{2010}^{H}\;=\;0.60$. Isolation indices are as follows, ${\text{I}}_{1990}^{B}\;=\;0.82$, ${\text{I}}_{1990}^{H}\;=\;0.48$, ${\text{I}}_{2000}^{B}\;=\;0.79$, ${\text{I}}_{2000}^{H}\;=\;0.532$, ${\text{I}}_{2010}^{B}\;=\;0.73$, ${\text{I}}_{2010}^{H}\;=\;0.55$. Information theory indices are as follows, H_1990_ = 0.54, H_2000_ = 0.47, H_2010_ = 0.42. Subscripts indicate Census years and superscripts indicate the minority group with respect to which an index was calculated; *B* for blacks and *H* for Hispanics. These indices represent an extreme compression of information in comparison with diversity maps, they can give only a very rough indication of diversity conditions and how they are changing.

Spatial versions of D, I, and H have been developed [27, 29–31] using a local neighborhood constructed around a focal Census unit to calculate the values of these indices. With such modification different values of indices are assigned to different units making the result mappable. Examples of such maps for the Chicago area can be found in the recent by paper by [15] (figures 5a, c, d and 7a, b, c in their paper). Each index requires its own map; thus, if we consider three indices listed above and two minority groups (Blacks and Hispanics), we need five maps to depict the racial condition in this area in a single year. Although these maps have their own merits, they don’t convey the racial conditions as clearly as our diversity maps do. In an another recent paper by [17] a map of different communities (for the Los Angeles area) was constructed by clustering multi-scale segregation profiles [24]. For a single year their map has a functionality similar to our diversity maps, but the method cannot be applied for multi-year comparison because it will yield differently-defined clusters for different years.

The only approach to analyze diversity in a way similar to what we advocate here has been done in [14]. Their approach also results in a map of categorized communities. However, their maps are tract-based and thus are subject to the issues summarized in Table 1. Their maps for the Chicago area (and other major MSAs) are available for viewing at http://mixedmetro.com/ making a direct comparison to our Fig 3 possible. Because Mixed Metro maps are tract-based they lack detail (for example, they don’t differentiate between inhabited and uninhabited areas). Although they can be used to get a general idea about the dynamics of diversity, they cannot be used for the type of quantitative analysis we will introduce in the next section because this requires grid-based maps.

## Quantitative analysis of diversity landscape

A diversity grid not only provides a compelling visualization of the overall racial/diversity condition in an area of interest (hereafter referred to as a *site*), but it also can be used as an input (instead of several race sub-population grids) for quantitative analysis of “diversity landscape.” First, that a diversity map is a categorical raster. Closer inspection (see the lower row of panels in Fig 3) reveals that a diversity map consists of a large number of *patches*, where a patch is defined as a contiguous group of same-color (same community category) cells. All patches of the same color indicate an area inhabited by a given community. Thus, the diversity grid has the same data format as a *landscape* in the field of landscape ecology [54]. Quantitative analysis of ecological landscapes is a highly developed topic [32, 33] with decades of development and an established software package (see below) for quantitative analysis. All this experience and methods of analysis can be carried over to diversity landscapes without modification.

We now give a brief introduction to the methodology of landscape metrics while changing original ecological nomenclature to fit the diversity context. The pattern of diversity in a site emerges from the composition and spatial configuration of community patches. It is an indicator of decades of socio-economic processes at this site. Accelerated changes in diversity patterns point to a transition from one process to another. For example, increasing areal coverage by medium diversity and high diversity communities at the expense of low diversity communities signals the increasing acceptance of multi-racial society. A quantitative comparison between landscapes at two different sites, or at the same site at two different times, is performed using landscape metrics (LMs) [32, 33]. LMs are algorithms that quantify a variety of spatial or non-spatial characteristics of a landscape patterns. A large number of different metrics have been developed and collected in a single computer program FRAGSTATS [33]. FRAGSTATS is a de facto standard for calculation of landscape metrics and is freely available at http://www.umass.edu/landeco.

LMs can be grouped into those which describe a single community, we will refer to them as c-metrics (community-level metrics), and those which describe the entire landscape (all communities together), we will refer to them as l-metrics (landscape-level metrics). They can also be grouped into compositional and configurational metrics. Compositional metrics are not spatially explicit, they measure what communities are present in a site and in what proportions they occur without reference to where in the landscape they may be located [54]. Configurational metrics provide a quantitative description of the spatial arrangements of different communities within the site. They could be further divided with respect to the aspects of the spatial pattern they are describing.

Table 3 summarizes various types of LMs. Examples of metrics from each type are given together with the abbreviation of their names as used in FRAGSTATS so a reader can refer to FRAGSTATS documentation [33] for the definition of each metric. We also indicate whether a metric is an l-metric, c-metric, or could be used to describe both, an entire landscape and a single community.

**Table 3:** Summary of landscape metrics.

TYPE	EXAMPLES OF METRICS	DESCRIPTION
**Compositional**		
	*PLAND*—percentage of landscape inhabited by each community (c); *PR*—richness (l); *SHEI*—evenness (l); *LPI*—largest patch index (c,l); *SHDI*—Shannon’s diversity index (l)	describe the non-spatial properties of individual communities as well as properties of the entire diversity landscape
**Configurational**		
Area & Edge	*AREA_MN*—patch areas distribution (c,l); *GYRATE_MN*—radius of gyration distribution (c,l); *TE* –total edge (c,l);	describe the spatial *grain* of diversity landscape
Shape	*PARA*—parameter-to-area distribution (c,l); *SHAPE*—shape index (c,l); *FRAC*—fractal dimension index (c,l); *CONTIG_MN*—contiguity index distribution (c,l)	describe the geometric complexity and/or compactness of shapes of community patches
Aggregation	*AI*—Aggregation index (c,l); *CONTAG*—contagion (l); *IJI*—interspersion & juxtaposition index (l)	describe the texture of diversity landscape
Subdivision	*NP*—number of patches (c,l); *DIVISION*—landscape division index (c,l); *MESH*—effective mesh size (c,l)	describe the compositional makeup of diversity landscape
Isolation	*ENN_MN*—Euclidean nearest neighbor distance distribution (c,l); *CONNECT*—connectance index (c,l); *PROX_MN*—proximity index distribution (c,l)	describe the degree of spatial isolation between community patches
Contrast	*CWED*—contrast-weighted edge density (c,l); *TECI*—total edge contrast index (c,l)	describe the magnitude of contrast (difference) along boundaries between different communities

Compositional metrics are similar to what is used in demography but they pertain to the size of areas instead of the number of people. Thus, *PLAND* gives the percentage of a site’s area inhabited by a given community rather than the percentage of total population belonging to this community. *SHEI* is just a standardized entropy but again applied to areas instead of racial groups. *LPI* has no equivalent in demography; it’s the ratio of the area of the largest patch in the site and the site’s total area. Configurational metrics assess the properties of spatial patterns and have no equivalents in demography. Just like compositional metrics, configurational metrics relate to areas (community patches) rather than population counts.

### Landscape metrics for the Chicago site

To illustrate how landscape metrics can quantify information contained in a diversity map we calculated selected metrics for the Chicago site (using the broader spatial extent site). First, in Table 4, we give the values of six popular landscape-level metrics (l-metrics). They describe the diversity landscape as a whole without subdividing it into specific communities.

**Table 4:** Landscape level metrics for the Chicago site.

Year	*NP*	*LPI*	*AI*	*MESH*	*CONTAG*	*IJI*	*TECI*
1990	4143	5.1	94	1593	74.5	42	11.6
2000	7469 +80%	4.9 -4%	92 -2%	1179 -26%	66.9 -10%	45 +7%	14.8 +28%
2010	12833 +72%	4.5 -8%	88 -4%	823 -30%	60.5 -9.5%	51 +13%	18.2 +23%

For 2000 and 2010 the values of these metrics are followed by a percentage change relative to the previous Census. The *NP* is the total number of community patches and it has been steeply increasing since 1990. This indicates that the site is disaggregating into a smaller, more intermingled mosaic of different communities. The *LPI* is the relative size of the largest patch (for example, the largest contiguous area inhabited by a single community in the Chicago site in 2010 was 4.5% of the entire populated area of the site). The *LPI* has been decreasing since 1990 which is consistent with the increase of *NP*. *AI* or Aggregation Index measures the tendency of the diversity landscape to aggregate into large patches; its range is between 0 (landscape divided into many small patches) and 100 (landscape aggregated into a single large patch). The value of *AI* has been decreasing since 1990 but it remains high, indicating that Chicago continues to be segregated despite changing toward increasing diversity. *MESH*—an effective mesh size, is an area-weighted mean patch size, or the size of the patch that can be accessed from a randomly chosen cell without leaving the patch. This is a measure of landscape subdivision and it has been steeply decreasing since 1990 which is consistent with the trends of *NP* and *LPI*. *CONTAG* summarizes the clumpiness of a diversity landscape and has a range between 0 (total disaggregation) to 100 (maximal aggregation). *CONTAG* shows the same trend as *AI* because they both assess the same property of a pattern. *IJI* measures the magnitude of interspersion of patch types; it also has a range between 0 (minimum interspersion) to 100 (maximum interspersion). It is expected to have an opposing trend to *AI* and *CONTAG* and, indeed, its value has been increasing since 1990. Finally, *TECI* measures the contrast (difference) between communities along boundaries between their patches. To calculate *TECI* an analyst must provide a matrix of dissimilarities between different communities, thus, for example, stating that the difference between a white dominated low diversity community and a black dominated low diversity community is high, whereas differences between medium diversity communities are smaller. The values of *TECI* have been increasing at the site due to the increased segmentation of all communities. Overall, these metrics quantify what is observed in Fig 3; in the last two decades the racial diversity in the Chicago area has been increasing, but, at a larger scale, the area remains segregated.

In Table 5 we list ten community-level metrics calculated for seven out of thirteen population density-aggregated communities: white low diversity (WL), white medium diversity (WM), black low diversity (BL), black medium diversity (BM), Hispanics low diversity (HL), Hispanics medium diversity (HM), and high diversity (Hdiv). The value of each metric is listed for years 1990, 2000, and 2010, respectively. Percentage changes of these values relative to the previous Census are also listed.

**Table 5:** Community level metrics for the Chicago site.

Metric	WL			WM			BL			BM			HL			HM			Hdiv		
*PLAND*	67	49 -27%	39 -20%	15	23 +35%	29 +26%	11	13 +18%	13 +0%	2.3	4.2 +83%	4.7 +12%	1.5	2.3 +53%	4.1 +78%	3.0	5.6 +87%	7.8 +39%	0.2	0.9 +350%	1.5 +67%
*NP*	116	1759 +1416%	2709 +54%	1528	2443 +60%	3203 +31%	211	471 +123%	699 +48%	432	795 +84%	1617 +103%	225	486 +116%	1031 +112%	439	772 +76%	2092 +171%	54	255 +372%	605 +137%
*LPI*	5.1	4.9 -4%	4.5 -8%	0.7	0.65 -7%	0.46 -29%	1.9	2.38 +25%	2.0 -16%	0.008	0.17 +2025%	0.23 +35%	0.36	0.4 +11%	0.5 +25%	0.26	0.4 +54%	0.28 -30%	0.06	0.13 +117%	0.1 -23%
*AREA_MN*	92	41 -55%	20 -51%	15	13.4 -11%	12.7 -5%	81	41 -49%	26 -37%	8.1	7.6 -6%	4.1 -46%	10	9 -10%	5.6 -38%	10.6	10.5 -1%	5.6 -47%	5.5	5.2 -5%	5.3 +2%
*AREA_AM*	2056	1862 -9%	1576 -15%	230	213 -7%	170 -20%	1550	1428 -8%	1145 -20%	42	56 -33%	61 +9%	193	227 +18%	224 -1%	104	167 +61%	78 -53%	39	47 +20%	32 -32%
*GYRATE_MN*	217	135 -38%	100 -26%	122	114 -7%	110 -4%	216	140 -35%	113 -19%	110	95 -14%	72 -24%	95	86 -9%	67 -22%	109	102 -6%	79 -23%	84	83 -1%	69 -17%
*GYRATE_AM*	1820	1655 -9%	1527 -8%	638	621 -3%	601 -3%	1784	1597 -10%	1486 -7%	305	329 +8%	317 -4%	540	630 +17%	616 -2%	441	527 +20%	376 -29%	252	273 +8%	238 -13%
*ENN_MN*	146	143 -2%	144 +0%	222	156 -30%	129 -17%	316	303 -4%	278 -8%	302	284 -6%	209 -26%	651	428 -34%	312 -27%	377	277 -27%	177 -36%	1724	557 -68%	366 -34%
*ENN_AM*	66	69 +5%	73 +6%	129	69 -47%	89 +29%	107	149 +39%	98 -34%	267	256 -4%	138 -46%	417	339 -19%	177 -46%	283	143 -49%	134 -6%	770	478 -38%	269 -44%
*AI*	96	95 -1%	92 -3%	90	89 -1%	86 -3%	96	95 -1%	92 -3%	87	87 +0%	82 -6%	90	90 +0%	85 -6%	88	88 +0%	82 -7%	88	84 -5%	81 -4%

*PLAND* is defined in Table 3. *NP*, *LPI*, and *AI* are equivalents of l-metrics of the same names but applied only to a single community. *AREA* is the area of a single patch in units of hectares (ha); 100 ha = 1 km^2^. Distribution of the values of *AREA* calculated for all patches in a given community can be described by statistics such a mean, standard deviation, etc. In Table 5 we list two statistics, *AREA_MN* which is a mean, and *AREA_AM* which is an area-weighted mean. The distribution of the values of *AREA* may be highly skewed with few large patches and a large number of small patches. However, a cumulative area (and, by proxy, also the cumulative number of the inhabitants) of these few large patches may be larger than the cumulative area of many small patches. Thus, *AREA_MN* may not reflect the perception of most of the inhabitants, hence the need for *AREA_AM*.

*GYRATE*, measured in units of meters, is “the radius of gyration” of a single patch—the average distance between a patch centroid and all its constituent cells. It is a measure of the average distance an inhabitant can move within a patch before encountering another community. As with *AREA*, we list the mean and the area-weighted mean of *GYRATE* values. Note that *GYRATE_AM* is a “correlation length”—a measure of the physical connectedness of the diversity landscape. *ENN*, measured in units of meters, is the Euclidean nearest neighbor distance—the shortest straight-line distance between a given patch and its nearest patch belonging to the same community. We list the mean and the area-weighted mean of *ENN* values; both are measures of isolation. Note that in Table 5 the values of *ENN_AM* are smaller then the values of *ENN_MN*, meaning that large patches are surrounded by nearby small patches.

The spatio-temporal evolution of each community at the Chicago site during the period of 1990–2010 can be observed directly from diversity maps (Fig 3) and it can be quantified using the metrics listed in Table 5.

The WL community has been declining. Its habitat shrank (declining values of *PLAND*) and it fragmented into a larger number of smaller patches (increasing values of *NP* and decreasing values of statistics of *AREA*). However, in 2010, it was still the largest community with a habitat that was well connected (small values of *ENN* statistics) and strongly aggregated (large values of *AI* and *LPI*). The WM community—a product of the decline of the WL community—was on the rise. Interestingly, despite its expansion, the WM habitat had increased its segmentation (increasing values of *NP* and decreasing values of *AREA* and *GYRATE* statistics). This is because the WL community converts into the WM community throughout its habitat forming small patches of WM while at the same time fragmenting WL into smaller patches. Thus, increased segmentation may be a sign of both, the decline and rise of a community, depending on the context of the change.

The evolution of the BL community habitat was different during the 1990s and 2000s. During the 1990s the area (*PLAND*) and the core (*LPI*) of the BL habitat had increased, while during the 2000s they had decreased. The habitat had fragmented during the entire 1990-2010 period but remains very well connected and aggregated. The BM community is mostly the product of erosion of the BL community, during the period of 1990-2010 its habitat and core had increased while the off-core parts of the habitat became more fragmented. It is similar to the dynamics between WL and WM communities.

The HL community habitat had experienced significant growth while becoming more connected which is supported by values of all metrics in Table 5. However, like all other communities it also become more fragmented. A habitat can increase its connectedness while becoming more fragmented by increasing the overall number of patches (measure of segmentation) while also increasing the size of large patches. The HM community is a product of the expansion of the HL community. It experienced a rapid growth during the 1990-2010 period. However, while during the 1990s the HM habitat consolidated (larger mean patch, increased connectedness, decreased isolation), this trend had reversed in to 2000s. Finally, the Hdiv community started very small but has experienced large growth. The first decade was a period of growth and consolidation, but a in the second decade the growth was accompanied by some segmentation.

## Summary and conclusions

Our goal in this paper was to present a comprehensive framework for visualizing and analyzing spatio-temporal dynamics of racial diversity in the entire conterminous U.S. We believe that all parts of the presented framework lower barriers for investigating issues related to segregation and diversity in the United States.

Using grids instead of aggregated units may seem like a radical departure from established practice, but their advantages (see Table 1) start to be apparent [16, 17, 20, 24]. Our U.S.-wide database of race sub-populations grids covering the last three Censuses frees researcher from constructing their own grids and, instead, allows them to focus on the analysis. As construction of the quality grid is a tedious work requiring specialized know-how, existing studies tend to concentrate on a single metropolitan area [17]. An ability to download already existing grid for any part of the U.S. makes practical to extend the analysis beyond the major metropolitan areas.

The set of diversity maps provides the quickest way to get informed about spatio-temporal dynamics of racial diversity in the U.S. End users, who are not specialists in the racial segregation/diversity field of research but need to know the present status and temporal trends of geographical distribution of different communities, will find diversity maps appealing. First, the maps require no numerical analysis—the information is conveyed in a lucid, visual manner. This is demonstrated in Fig 3 using the Chicago area as an example. Second, our web-based application SocScape provides an immediate and convenient access to this information anywhere in the U.S. and over the three last Censuses. In fact, by just browsing in SocScape, a user can get better informed about segregation/diversity situation in the U.S. than in any other way we are aware of.

Finally, our proposal to use diversity maps and landscape metrics instead of race sub-population data and demographic indices in order to numerically assess trends in segregation/diversity offers an interesting alternative to the customary manner of quantitative analysis. It follows from our underlying point of view that segregation/diversity is inherently a spatial phenomenon with a lot of methodological analogies to an another spatial phenomenon—a geographical distribution of land cover. Once the analogy between segregation/diversity and land cover is fully appreciated our proposed method of numerical analysis is a logical choice. As we demonstrated on the example of the Chicago area, landscape metrics can provide more numerical information about the diversity patterns than traditional indices, while being directly connected to spatial patterns of diversity.

Gridded diversity maps can also support other forms of analysis usually encountered in analyzing land cover change. One is a detailed assessment of change using transition matrices. A transition matrix counts and tabulates changes in the character of local communities within a site from one year to another. Transition matrices were first used by [14] and [37] to count the number of Census tracts that changed their diversity label between 1990 and 2000, and 2000 and 2010, respectively. However, by using grid cells instead of tracts as counting units, much more precise transition matrices can be calculated, including transitions in the number of people instead of the number of units. Transition matrices can also be used for producing a detailed maps of diversity change. For examples of analysis based on grid-based transition matrices see [44]. Another form of analysis supported by diversity maps is the prediction of future change in diversity landscapes. With diversity grids available for 1990, 2000, and 2010, an empirical transition model [55] could be built using 1990 and 2000 data and verified using 2010 data. Such a model could then be used to predict a diversity map in 2020 and checked versus an actual diversity map once 2020 Census data become available.
